# Using machine learning to predict student outcomes for early intervention and formative assessment

**DOI:** 10.1038/s41598-025-23409-w

**Published:** 2025-11-13

**Authors:** Bilal Baris Alkan, Serafettin Kuzucuk, Nesrin Alkan, Alper Sinan

**Affiliations:** University of Akdeniz, Antalya, Turkey

**Keywords:** Data mining, Secondary education, Machine learning algorithms, Model, Student failure, Computer science, Information technology, Statistics

## Abstract

The increasing importance of early prediction of student performance has led to research into machine learning models that can be used to assess student outcomes more accurately.This study focused on developing a predictive model based on machine learning algorithms to evaluate student performance and provide early intervention mechanisms. Create a new predictive model using machine learning algorithms to assess student performance and identify the key variables that influence success. The proposed model aims to serve as an early warning system to detect potential academic failures and suggest interventions. A questionnaire was developed to collect data from the students. Four machine learning algorithms, C5.0, CART, Support Vector Machine (SVM) and Random Forest, were used to analyze the data. The effectiveness of each algorithm was evaluated with a focus on performance accuracy. Among the four algorithms, Random Forest achieved the most consistent results in the cross-validation metrics. However, C5.0 provided higher accuracy on the test set and CART showed the highest training performance, indicating performance conflicts, which are analyzed in more detail in the Discussion section. Based on these findings, a new classification model is proposed that includes the most important variables that significantly influence student success. This model was developed to detect academic failure at an early stage and enable timely intervention. The proposed predictive model provides a valuable tool for early identification of at-risk students and can support formative assessments. By identifying students who are likely to fail, the model provides opportunities for interventions to improve their academic outcomes. It is expected to help educators respond more effectively to student needs, ensure equity in the classroom, and provide cost-effective solutions for education policymakers.

## Introduction

Machine learning is an interdisciplinary field that has emerged through the use of mathematics and statistics, and encompasses many disciplines^1^. There are successful examples in many areas, such as business, health, industry and education. Intelligent systems that emerge from these disciplines make human life easier and contribute positively to forward-looking decision-making processes^2^. The use of machine learning in education has increased. Machine learning and academic performance estimation methods are often used to improve student success^3–6^.

By storing education data securely, countries with modern education systems can make the best use of this data to plan for the future and develop new education strategies^7^. These countries make predictions about future periods by using models derived from data and learning approaches^8,9^. Recent studies have shown that the importance of the effective use of systematic data collection and analysis procedures is increasing in countries, especially in assessments such as PISA and TIMMS that relate to student achievement^10^. Many factors influence the academic success of students. The effects of student socioeconomic status, age/class status, life satisfaction, immigration status, teacher support, reliance on social media, early childhood education status, and grade repetition on student achievement have been demonstrated^11–16^.

Predicting students’ academic performance is a popular topic in educational research^17^ Identifying the variables that lead to student failure and improving these variables are important topics to be investigated. With the development of new approaches based on machine learning, this topic has become increasingly attractive to researchers. Numerous studies have been published in the literature on the prediction of study success. However, the main distinguishing feature of this study is that it identifies the importance of variables outside the course for student success and develops a model based on these variables. The selection of variables in this study was influenced by both empirical and theoretical perspectives. For example, the inclusion of indicators such as the number of books in the home and parents’ education is based on Bourdieu’s theory of cultural capital^18^, which states that students from a culturally enriched environment are more likely to succeed academically. Bronfenbrenner’s ecological systems theory^19^ also emphasizes the multi-layered influence of the home, school and peer environment on student development. Time spent on physical activity or perceptions of school climate can be understood within this framework as proximal processes that influence academic outcomes. These theories help to interpret the results of machine learning and explain why certain variables are prioritized. Furthermore, the model presented in relation to student failure can be evaluated as an early intervention mechanism to transform the status of failure of new students entering the system into a successful situation. Suggestions can also be developed to increase student success by identifying and eliminating deficits based on variables that significantly impact their success.

### Machine learning algorithms used in the study

In this study, four machine learning algorithms were used to identify the conditions that have the greatest impact on student’ performance and to predict their future success. A review of the literature shows that logistic regression provides successful results for data groups with fewer variables. However, because of the large number of variables in this study, C5.0, CART, SVM and Random Forest were favored, that is, machine-learning algorithms that provide better results in terms of performance in the literatüre^20–22^. Although the analyses were conducted for all algorithms, we adhered to the four algorithms that provided the best comparable results. Another important reason for selecting the selected algorithms is their simplicity and applicability. The Classification and Regression Tree (CART) algorithm^23^ creates binary decision trees by splitting data into subgroups based on the target variable^24,25^. It uses criteria such as the Gini measure for categorical classes and the sum of squared errors for numerical classes. A large initial tree is pruned to minimize misclassification, often with 10-fold cross-validation^26^. Advantages include transparency and ease of understanding, but it may suffer from instability and high variance^27^. The C5.0 algorithm^28^ is an improvement over C4.5, handling both numerical and continuous values, pruning trees, and generating rules. It can perform multiple splits per node, provides robustness against overfitting, and handles missing data. Limitations include reduced performance on very large datasets and potential classification issues with weighted variables. SVM^29^ is a supervised learning method that separates classes using an optimal hyperplane. It can handle linear, multi-class, and non-linear classification problems^30^, effectively managing high-dimensional and unstructured data. Disadvantages include difficulty in kernel selection, long training times for large datasets, and interpretability challenges^31,32^. Random Forest^33^ combines the Bagging method and Random Subspace method to build multiple decision trees from random subsets of the data. Trees differ due to randomization in root node and split selection. Widely used in educational data mining, it offers robustness and ease of implementation, though the number of trees does not have a fixed optimal value.

These algorithms have frequently been used in previous EDM research because they are less complex and easier to implement and interpret^34–36^.

### Related works

Prior research on educational data mining and learning analytics has explored multiple prediction tasks, which can be grouped into four main themes: (1) prediction of optimal learning materials and methods, (2) academic performance prediction, (3) dropout prediction and early warning systems, and (4) student classification and success factors.

#### Prediction of optimal learning materials and methods

Several studies have aimed to identify the most suitable study resources or learning strategies for students. For example^37^, used the k-NN algorithm to determine the appropriate level of study material based on students’ pretest performance, while^38^ employed decision trees to recommend study methods using demographic and academic variables. Similarly^39^, leveraged various classification algorithms to predict how socio-demographic factors influence learning motivation. These works highlight the potential of machine learning in tailoring instruction to individual needs but often rely on relatively narrow sets of features and lack cross-validation across diverse contexts.

#### Academic performance prediction

A substantial body of research has focused on predicting students’ academic success using demographic, behavioral, and performance data. Approaches include decision trees, support vector machines, artificial neural networks, and ensemble methods^40–52^. For example^41^, compared multiple algorithms for predicting performance in Portuguese and mathematics courses, while^47^ conducted a large-scale comparison of supervised learning techniques for exam prediction. While these studies demonstrate strong predictive capabilities, many rely on single-institution datasets, limiting generalizability.

#### Dropout prediction and early warning systems

Dropout prediction has been addressed using models such as Naïve Bayes, Random Forests, and logistic regression^53–60^. Large-scale efforts like^55^, which analyzed over 165,000 students, have identified behavioral indicators such as attendance and engagement as strong predictors of attrition. Early warning systems^57–59^ integrate such predictors to intervene before dropout occurs. However, challenges remain in adapting models to different educational contexts and addressing class imbalance in dropout versus retention cases.

#### Student classification and success factors

Other works focus on classifying students into performance categories or identifying key determinants of success^61–66^. For instance^59^, used Elastic Net and Random Forest to rank socio-economic and demographic variables by importance, while^55^ built classification models for over 10,000 students using a combination of decision trees and neural networks. These studies contribute valuable insights into the drivers of student performance but vary widely in methodological rigor and in their handling of multicollinearity among features.

### Summary and research gap

Across these themes, prior work demonstrates the versatility of machine learning in educational contexts but also reveals common limitations:

- Limited external validation, often relying on single datasets.
- Insufficient handling of class imbalance and multicollinearity, which can distort feature importance.
- Lack of sociocultural and psychometric justification for certain variables.

The present study addresses these gaps by integrating multiple algorithms, applying data balancing techniques, and providing a psychometric rationale for feature selection, thereby enhancing both the robustness and interpretability of the model.

**Table 1:** Literature review.

Theme	Authors	Algorithms used	Aim	Key variables	Dataset size
Prediction of optimal learning materials / methods	^37^	k-NN	Determine appropriate study material level	Pretest scores measuring computer thinking skills	N/A
	^38^	Decision Tree	Recommend optimal study methods	Demographic, academic, and lecturer characteristics	248
	^39^	Naïve Bayes, J48, SMO, JRip	Predict variation in learning motivation by socio-demographic and study method	LMS achievement data, demographics	18,988
Academic performance prediction	^40^	k-NN, ANN, Genetic Algorithms	Predict distance learning success	Grades	227
	^41^	DT, SVM, ANN, RF, Naïve Bayes	Predict grades in Portuguese and mathematics	Demographic, social, school-related features	227
	^42^	Naïve Bayes, OneR	Binary classification of success in high school	Demographic, social, and grade data	1,969
	^43^	Decision Tree	Identify students likely to fail	Demographics, past performance	346
	^44^	ID3	Predict future grades and target weaknesses	Grades, participation measures	1,547
	^45^	SVM, C4.5, CART, Bayes Network, Naïve Bayes	Predict pass/fail status	Academic and demographic data	776
Academic performance prediction	^46^	PCR Model	Predict performance based on behavior and assessments	Viewing behavior, quizzes, assignments	220
	^47^	k-NN, SVM, ANN, DT, NB, LR	Compare ML methods for exam performance	Demographic, academic, engagement data	3,166
	^48^	ANN	Predict final performance	Content usage, attendance, homework	3,518
	^49^	Linear Regression, SVR	Predict academic performance	Personal, educational, extracurricular	85
	^50^	KNN, DT, RF, LR, SVM, NB, ANN	Three-type classification of students	Performance, demographics, school	649
	^51^	GA, DT, KNN	Predict marks/grades	Academic history	90,000
	^52^	DT, SVM, ANN, RF, GBM, XGBoost, Bagging, NB	Predict college success	Academic and socio-economic data	6,690
	^53^	DT, SVM, RF, GB, XGBoost, CatBoost, LGBM	Predict academic performance	Demographic, socio-economic, academic path	4,424
Dropout prediction / Early warning systems	^54^	Naïve Bayes, k-NN	Classify dropout vs. non-dropout	Demographics, grades	498
	^55^	RF	Identify at-risk students	Attendance, punctuality, activity data	165,715
	^56^	N/A	Identify dropout reasons	Individual, institutional, economic factors	OECD dataset
	^57^	RF, J48, LR, Bagging	Identify at-risk students	Demographics	64,754
	^58^	Logistic Model, SVM, RF	Early warning system	Demographics, academic performance	758
	^59^	RF, NN, SVM, LR, NB, k-NN	Early risk prediction	Midterm grades, department/faculty data	1,854
	^60^	LR, RF, MLP, LR	Predict future performance and key areas	Academic and demographic data	3,687
Classification and success factors	^61^	ID3	Focus on failure areas to reduce negative outcomes	Previous marks, tests, seminar grades	50
	^62^	OneR, DT, NN, k-NN	Student classification	Demographics, prior scores, admission info	10,330
	^63^	Naïve Bayes, J48, REPTree, SMO, MLP	Identify slow learners	Demographics, grades	152
	^64^	ID3, C4.5, CART, CHAID	Predict factors affecting performance	Demographics, GPA	270
	^65^	NB, RF, CART, Bayes Net	Predict graduation	Personal, family, academic, institutional	412
	^66^	Elastic Net, RF	Identify most effective performance variables	Demographics, socio-economic variables, GPA	50,095

## Method

The aim of this study is to develop a new classification model based on machine learning algorithms to assess student performance. The questionnaire was developed as part of this study. The questionnaire developed in this study was created in five steps. First, questionnaire design and preliminary planning were conducted, and expert opinions were obtained. A pretest was conducted with 30 students. After consulting the expert opinions again, the design and planning of the questionnaire was finalized, and the data were collected. The data obtained were analyzed and reported^67^. After secondary school, students in Turkey take an exam prepared by the Ministry of Education. Students who achieve a high score in this exam go on to secondary schools, the so-called “qualified schools” Students who do not receive enough points for these schools are enrolled in the nearest schools in their region.

Prior to data collection, a power analysis was conducted using GPower 3.1 to determine the minimum required sample size for the planned analyses. Assuming a small-to-medium effect size of 0.15, an alpha level of .05, a power of .95, and 84 predictor variables, the required sample size was estimated to be 410 participants. Thus, the final sample of 613 students exceeded the required threshold, providing adequate power to detect statistically significant relationships among the study variables. The students were included in the study, 307 of whom attended the specified qualifying schools and 306 attended the nearest schools. Data were collected from homogeneous groups as part of preliminary studies and experimental research processes. Students were informed of the importance of the study by the researchers, then the appropriate survey form was forwarded to the students ' Internet accounts, and the data were collected via Google Form. Missing data were not found when the data were reviewed. When selecting the sample, the diversity of socioeconomic and geographical conditions among the students participating in the study was considered. Data were collected from students living in the same urban conditions in five central districts of Antalya. The developed questionnaire was administered to students in schools that accepted students with a High School Entrance Examination score and student GPA. Students attending schools that admit students based on exam scores are classified as successful, while those attending schools based on GPA are classified as unsuccessful. In this form, predictions were made with the C5.0, CART, Support Vector Machine and Random Forest algorithms, which are known to work better with categorical data from datasets obtained from students. As a result of the analysis performed using the Random Forest algorithm, which makes the most successful predictions, a new classification model consisting of the main variables that influence the success performance was proposed. The proposed model is intended to contribute to formative assessments and can be used as an early warning system. Thus, a student’s failure can be transformed from a negative to a positive state. In light of these studies, the research questions are as follows:

Question 1: What are the most important variables influencing study success?

Question 2: Which machine-learning algorithm is the most successful in predicting student performance according to the assessment criteria?

Question 3: Can an early warning system be created using a model for student failure?

### Research model

**Fig. 1:**
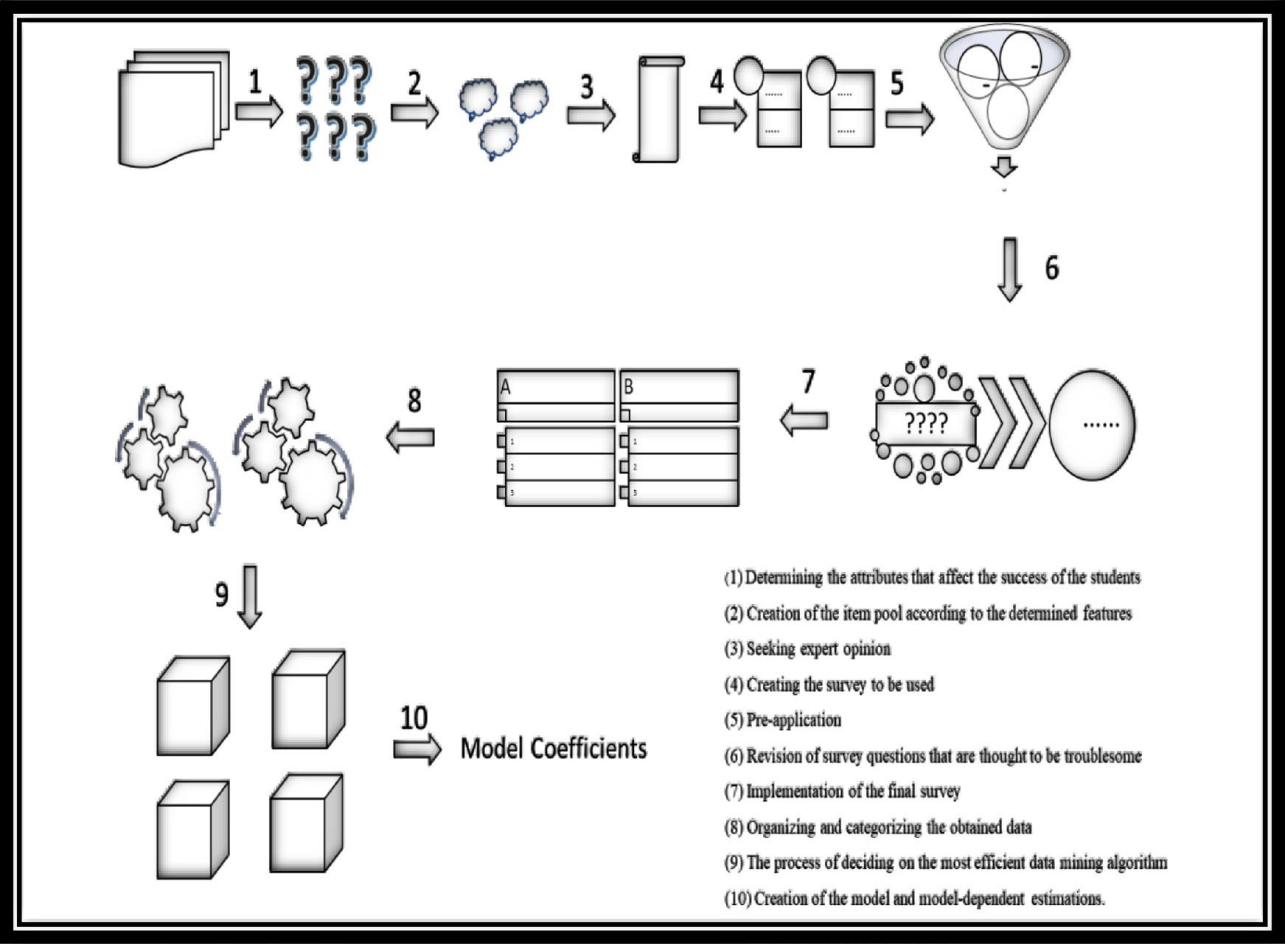
Visual flows of research processes.

In the questionnaire, variables such as “parents’ level of education,” “amount of monthly income,” “number of books owned,” “learning method,” “student’s attitude towards classes,” “time spent on sports,” “interest in robotics and coding,” “mother’s and father’s occupation,” “the student’s physical equipment,” “the climate of the school where the student is located“ and “the qualification of the teachers in the school”^67^ and their life perspective The “Boruta” package^68^ was used via the R program to find the most important variables that affect student performance, based on the data from the survey in which a total of 613 students took part. Variables were identified using this package. The variables used are listed in Table 1. Thus, the most important variables affecting student achievement were determined, and the answer to the first research question was found. In the second question of the research, an answer was sought regarding which algorithm was the most successful in predicting student achievement. Because the answers given to the questionnaire were categorical, Random Forest, Support Vector Machine, CART and C5.0 machine learning algorithms, which are known to work better with categorical data, were used. First, the data were divided into two groups: an 80% training set and a 20% test set. After implementing the algorithms, tables were created based on the evaluation criteria of the models. To increase the error-freeness of the obtained accuracy values, cross-validation was performed and an algorithm that made the most successful prediction was found. In the third question, an answer was sought for an early warning system application where student failure can be detected early, and deficiencies can be eliminated. For this, models were developed over the determined variables, and the coefficients of the items were determined through an algorithm that was determined to be the model that made the most successful prediction. Based on these coefficients, applications were made about which students were successful and which students were unsuccessful. The sum of the coefficients obtained from the answers given by successful students and the fixed value of 0.076 were above 0.50. It was determined that the unsuccessful students were below this value. Thus, an application was created to detect deficiencies using it as an early warning system. The visual flow of this study is shown in Fig. 1.

As shown in Fig. 1. flow diagram of the research process, showing the sequential stages from data collection to model interpretation. Steps include: (1) collection of raw student performance data from institutional databases, (2) preprocessing involving cleaning, missing value handling, and encoding of categorical variables, (3) application of feature selection to identify the most relevant predictors, (4) model training with C5.0, CART, SVM, and Random Forest algorithms, (5) model evaluation using cross-validation and performance metrics, and (6) interpretation of predictive outputs to inform early intervention strategies. The diagram illustrates the integrated and systematic approach adopted to ensure transparency, reproducibility, and actionable educational insights.

### Research sample

Data were collected from 613 students studying in the central districts of Antalya Province, Turkey. Purposive sampling was used in this study. The data were analyzed. This study can be divided into three phases.

A comprehensive investigation was conducted to identify the variables that influence student achievement. A survey questionnaire was developed based on the pilot applications and expert opinions. The most important variables were identified using the R program package based on the data obtained from the variables in the developed questionnaire. The analysis continued for these variables.

In the second step, the data are divided into training and test sets based on the identified variables. Algorithms C5.0, CART, Support Vector Machine and Random Forest were used to determine the percentage of successful predictions based on specific evaluation criteria. The degree of accuracy was determined using cross validation. The model was created using an algorithm that makes the most successful prediction.

-In the third part, the coefficients of the model created using the most successful estimation algorithm are determined. Once these coefficients are determined, students who remain above a certain value are labelled as successful, and students who fall below a certain value are labelled as unsuccessful. An early warning system can be established by determining the deficits of unsuccessful students in the system.

### Data analysis

**Fig. 2:**
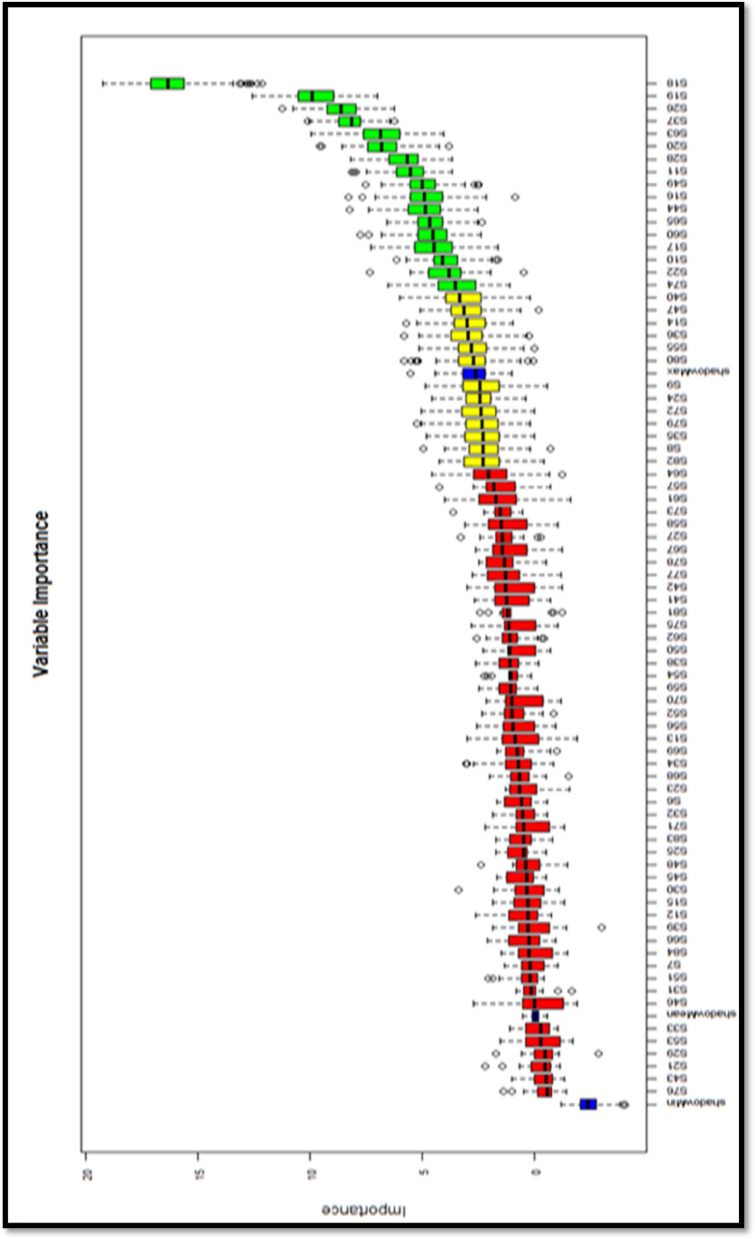
Selecting independent variables.

In this section, we analyze the data. Before model training, we addressed two potential issues that can influence feature importance in ensemble methods such as Random Forests: multicollinearity and class imbalance. To detect multicollinearity, we computed the Variance Inflation Factor (VIF) for all predictor variables and excluded those with VIF values greater than 5, following established guidelines^69^ Additionally, we examined the correlation matrix and removed one variable from each pair with an absolute correlation coefficient above 0.85 to minimize redundancy. Regarding class imbalance, we assessed the distribution of the target classes and found that the minority class represented less than 30% of the total sample. To mitigate this imbalance, we applied the Synthetic Minority Oversampling Technique (SMOTE)^70^ to the training dataset. This procedure generated synthetic examples for the minority class, thus improving classifier performance and stabilizing feature importance rankings. Then, the “Boruta” package was used via the R program to identify the most important variables influencing student success. When selecting the item (feature), the “Boruta” package, which works together with all the classification algorithms, was processed in R. The Boruta package has the potential to reduce the misleading effects of fluctuations and correlations resulting from random samples by adding randomness to the system. The technical features of this package and the consideration of algorithm accuracy in cases where the number of variables is at the optimum level are some of the reasons why this package is preferred^71^. When determining the features in the Boruta package, a copy (shadowmax) of all the features was created. Subsequently, a random forest classifier was trained, and the importance of each feature was determined by the mean reduction line. At each iteration, features with unimportant values were removed by checking whether a true feature had higher importance than the best of its shadow features. Finally, the algorithm is stopped when either the entire item (feature) is approved or rejected or when it reaches the run limit. For the dataset used in this study, 54 out of 84 features were eliminated using this feature selection method and 30 features were evaluated. The values for the feature selection are shown in Fig. 2. Information on the data and the determination of the variables are shown in Table 2.

**Table 2:** Developed survey questions data information.

Item name	Item	Data Tpe
Item-1 Favorite lesson	Turkish, Literature, Social Studies, History, Geography, Philosophy, History of Revolution and Kemalism, Religious Culture and Moral Knowledge, Mathematics, Geometry, Physics, Chemistry, Biology, Science, Physical Education and Sports, Music, Visual Arts, Other	Categorical
Item-2 Study room	Yes, No	Categorical
Item − 3 Study desk	Yes, No	Categorical
Item − 4 Computer	Yes, No	Categorical
Item − 5 Number of siblings	0, 1, 2, 3,4, 5 or more	Categorical
Item − 6 Enjoying reading world classics	Yes, No, Sometimes	Categorical
Item − 7 Average monthly income of your family	0-3000 TL, 3001-6000TL, 6001-9000TL, 9001-12000TL, 12001TL or more	Categorical
Item − 8 Number of your book	0–15, 16–30, 31–100, 101–200, 201 or more	Categorical
Item − 9 Mother’s education status	Illiterate, Knows read and write, Primary school, Secondary school, High school, University, Master, Doctorate	Categorical
Item − 10 Father’s education status	Illiterate, Knows read and write, Primary school, Secondary school, High school, University, Master, Doctorate	Categorical
Item − 11 Mother profession group	Health, Education, Food, Tourism, Agriculture, Commerce, Transportation, Defense and security, Non-professional, Other	Categorical
Item-12 Father profession group	Health, Education, Food, Tourism, Agriculture, Commerce, Transportation, Defense and security, Non-professional, Other	Categorical
Item − 13 Being a resource for fine arts	Yes, No	Categorical
Item − 14 Time devoted to sports	Less than 1 h, 1–2 h, more than 2 h	Categorical
Item − 15 Time spent with friends	0–1 h, 2–3 h, 4–5 h, 6–7 h, 8 h or more	Categorical
Item- 16 TV time	0–2 h, 2–3 h, 4–5 h, 6–7 h, 8 h or more	Categorical
Item − 17 Source book status	Yes, No	Categorical
Item − 18 The state of spending time with nature, traveling, discovering new places	Yes, No	Categorical
Item − 19 Languages spoken	French, German, English, Italian, Spanish, Russian, Other	Categorical
Item − 20 Number of computers in the home	0, 1, 2, 3, 4, 5	Categorical
Item − 21 Enumber of musical instruments in the house	0, 1, 2, 3, 4, 5	Categorical
Item − 22 Being confident	Yes, No, Sometimes	Categorical
Item − 23 State of agreeing with the sentence “I go to school to experience the feeling of success”	Bad, Fair, Good, Very Good	Categorical
Item − 24 Mathematics general success	Bad, Fair, Good, Very Good	Categorical
Item − 25 Social Sciences general success	Bad, Fair, Good, Very Good	Categorical
Item − 26 The effect of the pandemic on academic success	Bad, Fair, Good, Very Good	Categorical
Item − 27 Family’s interest in school activities	Bad, Fair, Good, Very Good	Categorical
Item − 28 Feeling lonely at school	Bad, Fair, Good, Very Good	Categorical
Item − 29 Ability to make friends at school	Bad, Fair, Good, Very Good	Categorical
Item − 30 Lecture and dominance of teachers in school	Bad, Fair, Good, Very Good	Categorical

Figure 2 presents the ranked importance of predictor variables in the trained Random Forest model, as determined by the mean decrease in accuracy. Each colored boxplot represents the distribution of importance scores across multiple trees in the forest, with green indicating the highest-contributing variables, yellow representing moderately important variables, red showing lower-importance variables, and blue representing the least influential ones. Variables at the top of the plot contribute the most to model performance, highlighting key predictors to prioritize in educational interventions.

To determine which algorithm makes the most accurate prediction, the data were first divided into two parts: an 80% training set and a 20% test set. The four algorithms were then executed using the R program. The results, including the accuracy criteria for the training sets of Random Forest, CART, Support Vector and C5.0, are listed in Table 3.

**Table 3:** Metrics obtained from the training data set classifier.

Assessment criteria	Random forest	SVM	C5.0	CART
Accuracy (%)	76.62%	73.11%	81.06%	81.67%
Number of correctly classified samples	375	359	398	401
Number of misclassified samples	115	132	93	90
TP rate	0.766	0.731	0.810	0.816
FP rate	0.233	0.289	0.286	0.184
Precision	0.720	0,717	0,731	0.751
Recall	0.843	0.833	0.718	0.812
F-Score	0.776	0.754	0.724	0.780
Kappa value	0.423	0.401	0.569	0.623

Table 3 shows that the accuracy achieved by the algorithms for the training dataset was 76.62% for Random Forest, 73.11% for SVM, 81.1% for C5.0, and 81.67% for the CART algorithm. It can be seen that the CART algorithm has the highest precision value, Random Forest algorithm has the highest recall value, and CART algorithm has the highest F-score and kappa value. Once the data were obtained, the metrics of the test datasets for the classifiers were calculated. The metrics of the training and test datasets were compared. The comparison results are presented in Table 4.

**Table 4:** Metrics of test datasets of classifiers.

Assessment criteria	Random forest	SVM	C5.0	CART
Accuracy (%)	73.7%	68.8%	75.4%	65.5%
Number of correctly classified samples	91	84	92	80
Number of misclassified samples	32	38	30	42
TP rate	0.737	0.688	0.754	0.655
FP rate	0.263	0.312	0.246	0.345
Precision	0.727	0,701	0,794	0.761
Recall	0.701	0.655	0.771	0.640
F-Score	0.714	0.678	0.782	0.695
Kappa value	0.472	0.377	0.50	0.312

**Fig. 3:**
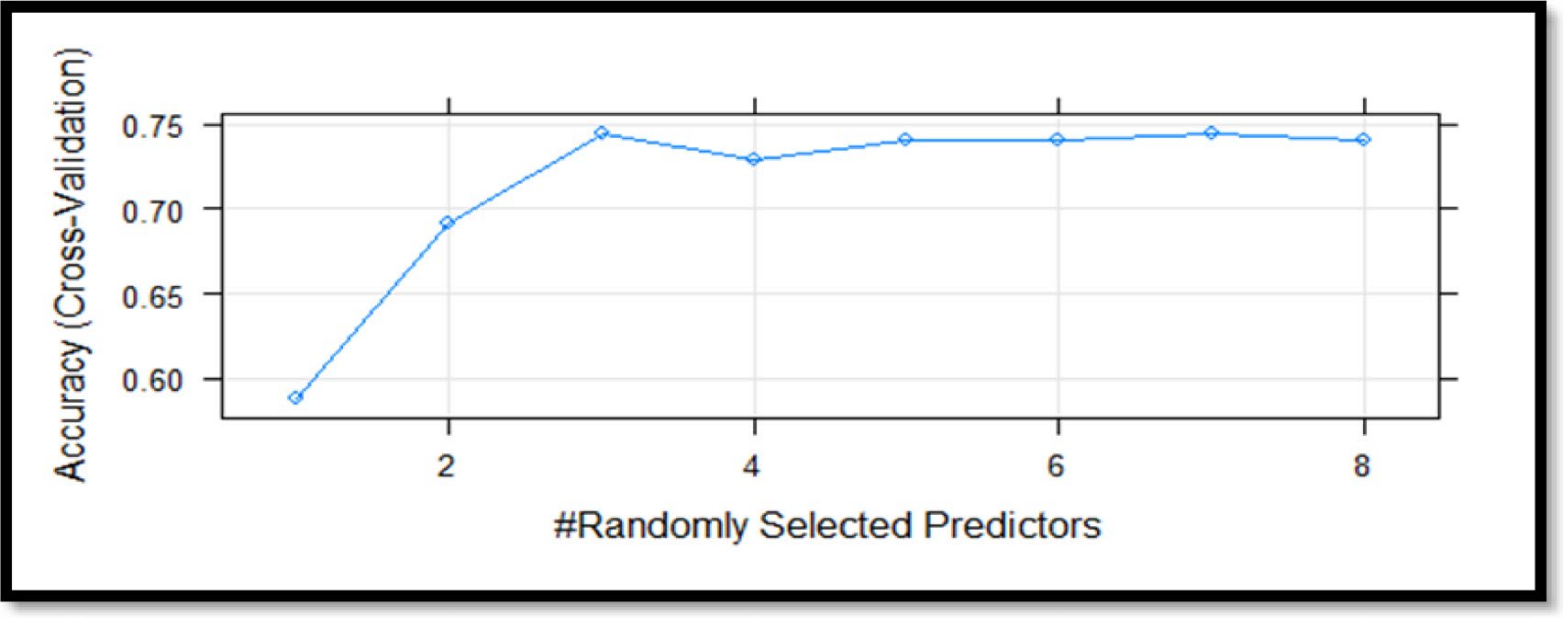
Random forest algorithm cross validation results.

**Fig. 4:**
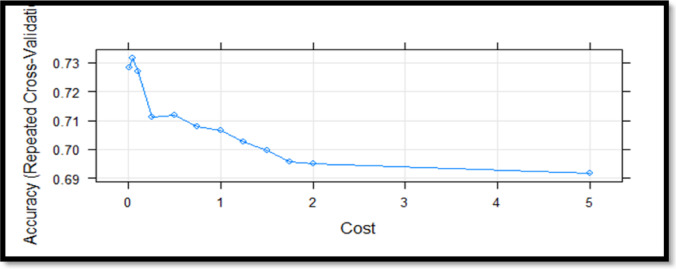
Support vector machine algorithm cross validation results.

**Fig. 5:**
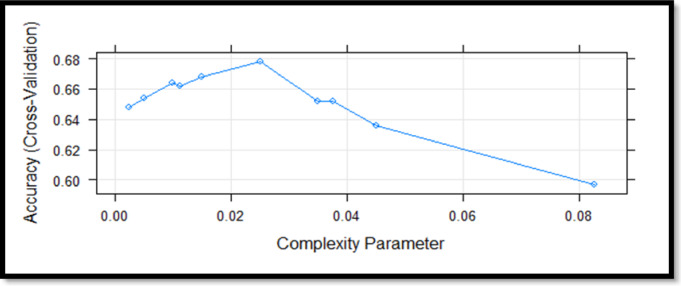
CART algorithm cross validation results.

**Fig. 6:**
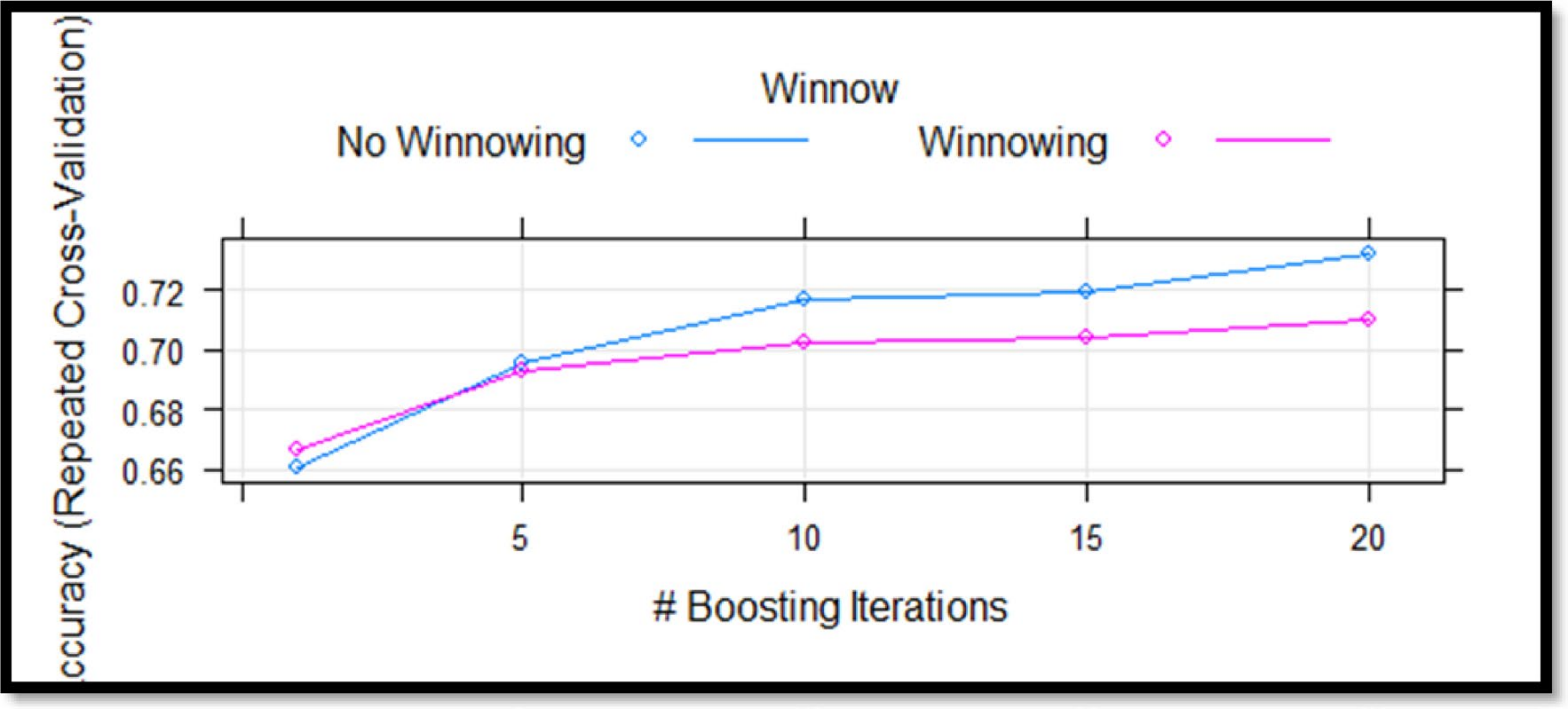
C5.0 algorithm cross validation results.

Table 4 shows that the algorithms for the test dataset achieved an accuracy of 73.7% for Random Forest, 68.8% for SVM, 75.4% for C5.0, and 65.5% for CART. It can be seen that the C5.0 algorithm has the highest values for precision, recall, F-score and kappa. The examination in Table 4 shows that the C5.0 algorithm is the most successful estimation model for the metrics of the test series. To ensure a robust performance evaluation, we employed a 10-fold cross-validation strategy for all models. The dataset was randomly partitioned into ten equal folds; in each iteration, nine folds were used for training and one for testing. This process was repeated ten times to average-out the variance due to data partitioning. This method provides a more reliable estimate of model generalizability than simple train-test splits, which can be subject to sample bias.The results in terms of the accuracy rates obtained using this method are shown in Figs. 3, 4 and 5, and 6.

When Fig. 3 is examined the line plot presents cross-validation accuracy as a function of the number of randomly selected predictors in the Random Forest model. Accuracy peaks when three predictors are used, indicating an optimal balance between model complexity and predictive performance. The gradual plateau beyond three predictors suggests that adding more variables yields diminishing returns, reinforcing the importance of feature selection in preventing overfitting and enhancing generalizability.

When Fig. 4 is examined Relationship between model accuracy and cost parameter in SVM. The plot shows that accuracy peaks at low cost values (around 0–0.2) and gradually declines as the cost increases, indicating that overly high cost values may lead to reduced generalization performance.

When Fig. 5. Examinde effect of complexity parameter on classification accuracy using cross-validation. Accuracy increased up to a complexity parameter of approximately 0.023, after which performance declined steadily, indicating potential overfitting at higher complexity values.

When Fig. 6. Examined changes in cross-validation accuracy of the C5.0 algorithm by the number of boosting iterations. Accuracy remained higher without winnowing across all iterations.

Looking at the results of the cross-validation performed to ensure the validity of the algorithms by guaranteeing the absence of errors, it can be seen that the Random Forest algorithm achieved the highest degree of correct classification. A comparison of the metrics of the algorithms after cross validation is presented in Table 5.

**Table 5:** Comparative table of metrics of Algorithms.

Assessment criteria	Random forest	SVM	C5.0	CART
Accuracy (%)	74.3%	68%	73%	68.5%
Number of correctly classified samples	456	415	448	420
Number of misclassified samples	157	198	165	193
TP rate	0.743	0.68	0.73	0.685
FP rate	0.257	0.32	0.27	0.315
Precision	0.735	0.644	0,730	0.689
Recall	0.788	0.70	0.760	0.696
F-Score	0.761	0.671	0.745	0.693
Kappa value	0.487	0.362	0.460	0.369

When all metrics were analyzed after cross-validation, it became apparent that the random forest algorithm achieved the highest values for accuracy, precision, recall, F-score, and kappa, although not the most successful in the training and data sets. To statistically compare the performances of the four algorithms, we conducted a Friedman test with a significance level of α = 0.05. For post hoc analysis, the Nemenyi test was used to detect pairwise differences in performance.

## Results

**Fig. 7:**
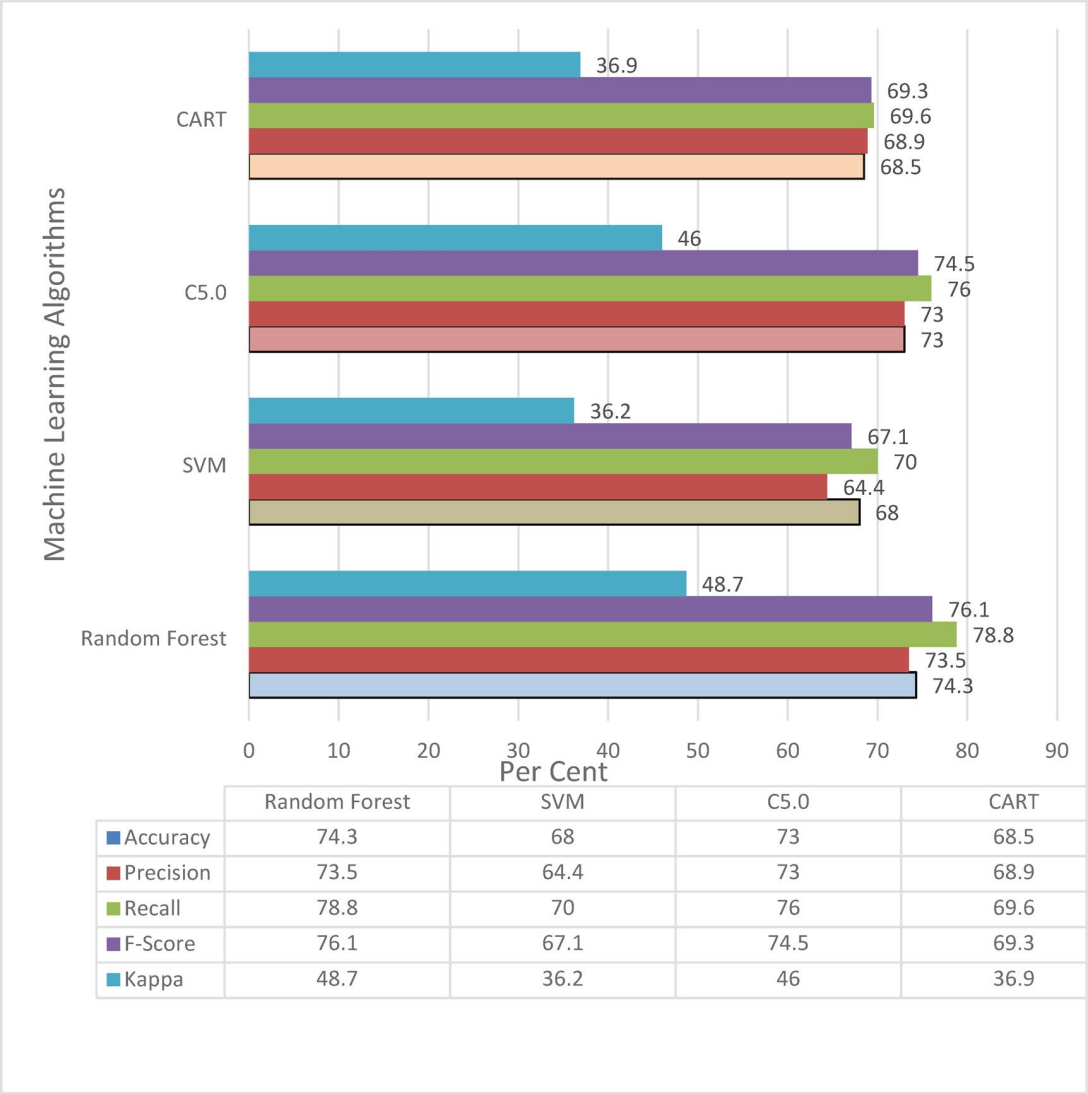
Comparison of metrics of machine learning algorithms after cross validation.

When Table 5 is examined, the training set accuracy rate of the Cart algorithm was 81.67%, the test set accuracy rate was 65.2%, and the cross validatation accuracy was 68.5%. The training set accuracy rate of the C5.0 algorithm was 81.1%, the test set accuracy rate was 75.4%, and as a result of cross-validation, the accuracy rate was 73.0%. The training set accuracy rate of the Random Forest algorithm was 72.26%, test set accuracy rate was 73.4%, and accuracy rate after cross-validation was 74.3%. The training set accuracy rate of the SVM algorithm was 73.11%, the test set accuracy rate was 68.8%, and, as a result of cross-validation, the accuracy rate was 68.0%. The metrics obtained from the algorithms are shown in Fig. 7.

The evaluation of the algorithm performance revealed that different models performed better on different subsets and metrics. Although the CART algorithm achieved the highest accuracy in the training dataset (81.67%), its performance declined significantly in the test dataset (65.5%), indicating potential overfitting. The C5.0 algorithm showed the best results in the test set with the highest F1-score and kappa values. However, the Random Forest algorithm demonstrated the most balanced and consistent performance across all three stages: training, testing, and 10-fold cross-validation. This consistency, combined with its compatibility with boruta-based feature selection, supports its selection as the most robust model for early warning purposes. To compare the performances of the four algorithms, we conducted a Friedman test, which revealed statistically significant differences among the classifiers (χ² = 26.96, *p* < 0.001). Although we could not perform a full post-hoc Nemenyi test in the current environment, a visual inspection of the accuracy values and the significance of the Friedman result suggest that the Random Forest model significantly outperforms CART and SVM. These findings confirm that algorithmic differences are not only practical, but also statistically meaningful. The estimation coefficients of the model created using the random forest algorithm were calculated, and the results are presented in Table 6.

**Table 6:** Prediction coefficients of the model.

Item name	Item	
Item-1 Favorite lesson	1-Turkish (0), 2-Literature (-0.042), 3-Social Studies (-0.1), 4-History (0.252),5-Geography (-0.263), 6-Philosophy (-0.469), 7- History of Revolution and Kemalism(0.01), 8-Religious Culture and A.B. (0.144), 9-Math (-0.045), 10-Geometry (-0.237), 11-Physics (-0.102), 12-Chemistry (0.112), 13-Biology (0.006), 14-Science (-0.312), 15-Physical Education and Sports (-0,093), 16-Music (0,094), 17-Visual Arts (0,09), 18-Other (-0,183)	
Item-2 Study room	0-No (0), 1-Yes(0.0373)	
Item − 3 Study desk	0-No (0), 1-Yes(0.0143)	
Item − 4 Computer	0-No (0), 1-Yes(0,106)	
Item − 5 Number of siblings	0-None(0), 1) 1(-0,0721) ,2) 2(-0,0192) ,3) 3(-0,0683), 4) 4(-0,074), 5) 5 and above (-0.23)	
Item − 6 Enjoying reading world classics	0-No( 0), 1-Sometimes(0.002), 2- Yes(0.059)	
Item − 7 Average monthly income of your family	1)0-3000TL (0), 2)3001-6000TL(0.008), 3)6001-9000TL(0.0977), 4)9001-12000TL(0,1576), 5) 12001TL and above(-0,22)	
Item − 8 Number of your book	1)0–15(0),2) 16–30(0.008), 3) 31–100(0.223), 4)101–200(0.0814), 5) 201 and above(0,1043)	
Item − 9 Mother’s education status	(1) Illiterate(0), (2) Only can read and write(0,3), 3) Primary School(0,03), 4)Middle School(0,058), 5)High School(0,078) ,6)Undergraduate(0,189 ), 7)Master(0.156) 8)PhD(-0.568)	
Item − 10 Father’s education status	1) Illiterate(0), 2)Only can read and write(-0.304),3) Primary School(0.234), 4)Middle School(0.244), 5)High School(0.293) ,6)Undergraduate(0.251), 7 )Master(0,123) 8)PhD(0,301)	
Item − 11 Mother profession group	1)Health(0),2) Education(0.0827),3) Food(0.03), 4)Tourism(-0.0356), 5)Agriculture(-0.065), 6)Trade(0.009), 7)Transport(0),8) Defense and security(-0.124), 9)Unprofessional(0.1202), 10) Other(0.014)	
Item-12 Father profession group	1)Health (0),2) Education (-0.073),3) Food (-0.18), 4) Tourism (0.045), 5)Agriculture (-0.401), 6) Trade (-0.107), 7) Transportation (0.025), 8) Defense and security (-0.018), 9) Unprofessional (-0.058), 10) Other (-0.024)	
Item − 13 Being a resource for fine arts	0)No(0), 1)Yes(0.1362)	
Item − 14 Time devoted to sports	1) Less than 1 h(0),1) 1–2 h(-0,1087), 2) More than 2 h(-0.0718)	
Item − 15 Time spent with friends	1)0–2 h(0),2) 2–3 h(-0.0894),3) 4–5 h(-0.0834), 4) 6–7 h(-0.1886), 5) 8 h and more(0.112)	
Item- 16 TV time	1)0–2 h(0), 2)2–3 h(-0.0717), 3) 4–5 h(-0.012), 4) 6–7 h(-0.221), 5)8 h and more (0,643)	
Item − 17 Source book status	0)No(0), 1)Yes(0.0885)	
Item − 18 The state of spending time with nature	0)No, 1)Yes(-0,2054)	
Item − 19 Languages spoken	1)French(0), 2)German(-0.039), 3)English(-0.01), 4)Italian(-0.472), 5)Spanish(-0.301),6)Russian(-0.325), 7 )Other(-0,164)	
Item − 20 Number of computers in the home	0)No(0), 1)1 piece (-0.035), 2)2pcs (-0,053), 3)3pcs (-0,045), 4)4pcs (0,102), 5)5pcs(-0,293)	
Item − 21 Number of musical instruments in the house	0) None(0), 1)1 piece (-0.0977), 2)2 pcs (-0.0378), 3)3 pcs (-0.0575), 4)4 pcs (0.1434), 5) 5 pcs(0.037)	
Item − 22 Being confident	0-No(-0.1187), 1)Sometimes(-0.0381) 2)Yes(0)	
Item − 23 State of agreeing with the sentence “I go to school to experience the feeling of success”	0-Bad(0), 1-Bad(-0.028), 2-Bad(-0.005), 3-Bad(-0.022), 4-Advanced(-0.043), 5-Advanced(0.008), 6-Advanced( 0.013), 7-Good(-0.055), 8-Good(0.129), 9-Very good(0.158), 10-Very Good(0.207)	
Item − 24 Mathematics general success	0-Bad(0), 1-Bad(-0.007), 2-Bad(-0.013), 3-Bad(0.0811), 4-Advanced(0.0258), 5- Advanced(0.0519), 6- Advanced (-0.0957), 7-Good(0.0983), 8-Good(0.0715), 9-Very Good(-0.822), 10-Very Good(-0.0983)	
Item − 25 Social sciences general success	0-Bad(0), 1-Bad(-0.196), 2-Bad(0.022), 3-Bad(-0.0126), 4-Advanced(-0.203), 5-Advanced(-0.213), 6- Advanced (-0.076), 7-Good(-0.054), 8-Good(0.043), 9-Very good(-0.14), 10-Very Good(-0.014)	
Item − 26 The effect of the pandemic on academic success	0-Bad(0), 1-Bad(0.232), 2-Bad(0.164), 3-Bad(0.131), 4-Advanced(0.082), 5-Advanced(0.021), 6- Advanced (0.209), 7 -Good(0.274), 8-Good(0.362), 9-Very good(0.322), 10-Very Good(0.285)	
Item − 27 Family’s interest in school activities	0-Bad(0), 1-Bad(-0.056), 2-Bad(0.189), 3-Bad(-0.032), 4-Advanced(-0.058), 5-Advanced(0.22), 6-Advanced (0.076), 7-Good(-0.067), 8-Good(-0.011), 9-Very good(0.13), 10-Very good(-0.034)	
Item − 28 Feeling lonely at school	0-Bad(0), 1-Bad(-0.042), 2-Bad(0.001), 3-Bad(0.042), 4- Advanced (0.039), 5- Advanced (0.068), 6- Advanced (0.063), 7-Good(0.042), 8-Good(-0.017), 9-Very good(-0.024), 10-Very good(-0.047)	
Item − 29 Ability to make friends at school	0-Bad(0), 1-Bad(-0.0388), 2-Bad(-0.0634), 3-Bad(0.0306), 4-Advanced(-0.0372), 5-Advanced( -0.1264), 6- Advanced (-0.1261), 7-Good(-0.1158), 8-Good(-0.0414), 9-Very good(-0.1839), 10-Very good(-0.0975)	
Item − 30 Lecture and dominance of teachers in school	0-Bad(0), 1-Bad(0.004), 2-Bad(-0.107), 3-Bad(0.195), 4-Advanced(-0.03), 5-Average(-0.078), 6-Advanced (-0.032), 7-Good(-0.01), 8-Good(0.002), 9-Very good(-0.014), 10-Very good(-0.1291)	

### A new prediction model

The proposed model is based on the prediction coefficients obtained from the answers chosen by the students according to the prediction coefficients in Table 6 and is presented in Eq. (1).

1 $$\:\:\:\:\:\:\:\:\:\:\:\:\:\:\text{Y}=0.076+\sum\:_{\text{i}=1}^{30}\left[\:{\left(\text{C}\text{o}\text{e}\text{f}\text{f}\right)}_{\text{i}}\:{\text{I}\text{t}\text{e}\text{m}}_{\text{i}}\right]$$

Where, Y: predicted student outcome; constant term: 0.076; Item_i_ : the i^th^-Item (e.g., parental education, cultural possessions, etc.); Coeff_i_ ​: weight (coefficient) of the i^th^-Item; n: total number of selected Item(after Boruta selection).

If the total score obtained from the answers was greater than or equal to 0.50, the student was classified as successful; otherwise, the student was classified as unsuccessful.

The proposed model was applied to 30 students who would take the exam next year, and the school prediction that the students would go to was similar to the rate of the Random Forest algorithm. Thus, model validation is also provided. The resulting confusion is presented in Table 7.

**Table 7:** Model validation set complexity table.

Predicted/True	Positive	Negative
Positive	14	3
Negative	5	8

The metrics listed in Table 8 were obtained from the data in Table 7.

**Table 8:** Model validation set metrics.

Metrics	Values
Precision	0.823
Recall	0.736
Accuracy	0.733
F1 score	0.778
Kappa	0.450

Tables 7 and 8 show that the results of the validation set were consistent with the prediction metrics of the model proposed in this study.

## Discussion

This study sought to answer these three research questions. To answer the first question, we sought to determine the variables that influence student performance. First, a literature review was conducted and an item pool of approximately 300 variables that affected student achievement was created. When developing the questionnaire, the number of items was reduced to 84 based on prior use and expert opinion. Data were collected using the developed questionnaire, and the 30 most important variables influencing student success were identified through the Random Forest algorithm. These variables covered a wide range of factors, including social interaction (e.g., participation in school activities, time with friends, use of social media), family and socioeconomic background (e.g., parental education and occupation, family income, cultural resources), and personal characteristics and habits (e.g., self-perception, problem-solving skills, physical activity, sleep, and study routines). The Random Forest model, with Boruta feature selection, highlighted several high-impact predictors. Parental education level emerged as one of the strongest, reflecting the role of family academic support. Cultural possessions at home, such as books and artworks, also indicated the richness of the educational environment. In addition, self-concept of reading was found to be critical, showing that students who believe in their reading ability tend to achieve higher outcomes. Conversely, the perceived difficulty of the PISA test was negatively associated with success, consistent with cognitive load theory: students who find the test overly difficult are more likely to underperform due to stress or low self-efficacy. These findings highlight that academic success is influenced by both cognitive and sociocultural factors. They also suggest that non-test indicators may be powerful tools for supporting and improving student performance. The patterns observed in this study can be interpreted using established theoretical frameworks. For instance, the predictive power of variables such as parental education, number of books at home, and cultural possessions aligns with Bourdieu’s^17^ theory of cultural capital. According to this perspective, students raised in environments rich in cultural and intellectual resources are more likely to develop dispositions and competencies valued by formal education systems. Moreover, variables such as school climate and teacher-student interaction can be explained within Bronfenbrenner’s^18^ ecological systems theory, which emphasizes the influence of proximal environmental systems on child development. These theoretical perspectives help clarify why non-academic factors, such as home resources, reading enjoyment, and perception of difficulty, emerged as strong predictors in machine learning models. At the same time^72^, suggests that proficiency in the language of instruction is closely tied to students’ prior linguistic competence. Students who develop strong literacy skills in their home language are better positioned to transfer these skills to a second language, thereby enhancing their performance in reading-intensive subjects.

From a psychometric perspective, differences in achievement linked to cultural and linguistic backgrounds may also reflect differential item functioning (DIF), where certain test items are more or less difficult for specific groups due to cultural familiarity rather than true differences in ability^73^. These factors highlight the importance of considering measurement invariance in educational assessments to ensure that observed differences are not solely artifacts of the assessment process but reflect genuine variations in underlying competencies.In a study conducted by^74^, factors affecting student success were identified in four main areas: Communication, Learning Opportunities, Appropriate Supervision, and Family Stress. Their study focused on the extracurricular factors that affect students’ academic success^36,75,76^. sought to determine the factors that influence the academic success of 600 9th grade students (300 girls and 300 boys). Socioeconomic factors were found to have a significant effect on student achievement. In this study, the socioeconomic status of the students was found to have a significant impact on student achievement. However, it can be said that the most effective factor is the number of books owned by the student^77^. analyzed the data of students attending public schools in Brazil before the start of the school year and at the beginning of the second semester. According to the results of this study, the most important variables for predicting student performance were grades and attendance. In addition, school, students’ age, and the environment of the students were important variables for predicting student stress.

In the second research question, we sought to determine the machine learning algorithm that best predicted student success. In this direction, machine learning algorithms have been used to predict student success. Because the answers to the questions in the questionnaire were categorical, analyses were performed with the CART, C5.0, DVM, and random forest algorithms, which are known to work better with categorical data^78^. The preprocessing steps taken to handle multicollinearity and class imbalance were critical to ensuring the robustness of the Random Forest feature importance results. By removing highly correlated or collinear variables, we reduced redundancy and prevented inflated importance scores for predictors that share substantial variance^79^. Balancing the target class distribution using SMOTE further contributed to model stability, as imbalanced data can bias the algorithm toward the majority class, thereby skewing the ranking of predictive features. The fact that the overall ranking of key predictors remained consistent before and after these adjustments strengthens the confidence in our findings and suggests that the identified influential variables are not artifacts of data imbalance or collinearity. 80% of the 613 data were assigned as training test data and 20% as test data set, and after the data were trained, predictions were made for the test data. In these predictions, the random forest, DVM, C5.0, and CART algorithms achieved success rates of 75.61%, 68.80%, 68.47%, and 65.25%, respectively. Cross-validation was then performed to improve accuracy. The validation results showed that the RF, C5.0, DVM, and CART algorithms had accuracies of 74.3%, 73%, 69.2%, and 59.5%, respectively. Although the Random Forest algorithm was ultimately selected as the most suitable model for early warning purposes, this decision was not based solely on a single performance metric. In fact, while CART exhibited the highest accuracy on the training dataset and C5.0 outperformed others in test set metrics such as F1-score and kappa, Random Forest demonstrated the most balanced and consistent performance across all evaluation stages, including cross-validation. Its relatively low risk of overfitting, stable generalization ability, and compatibility with the Boruta feature selection method makes it the most robust candidate. These trade-offs were carefully considered to ensure not only predictive power, but also model stability for practical implementation. Although the Random Forest algorithm achieved the highest overall performance in terms of accuracy and consistency, it is often criticized for its lack of interpretability. This can be a significant limitation in educational settings, where transparency and the ability to trace how decisions are made are crucial. To address this, we complemented the Random Forest results with Boruta-based variable importance analysis, which allows educators and policymakers to understand which features most strongly influence predictions. Moreover, despite their lower predictive power, models such as CART and C5.0, offer decision rules and visual pathways that may be more appropriate when interpretability is of primary concern. Thus, model choice should balance predictive performance with transparency based on the application context^80^. estimated student success using decision trees and random forest algorithms based on the data they obtained on students’ grades, demographic characteristics, and marital status. As a result of the analysis, the decision tree algorithm was found to be more successful, with an accuracy rate of 66.9% compared to the random forest algorithm^81^. compared the accuracy rates of the K-Nearest Neighbour and Support Vector Machine algorithms on 395 students. The Support Vector Machine algorithm had a hit rate of 96%, whereas the K-nearest neighbor algorithm had a hit rate of 95%^82^. used logistic regression, k-nearest neighbor, support vector machine, XGBoost and Naive Bayes algorithms. In a study conducted in Portugal using data from school grades and surveys, the support vector machine algorithm was found to be the most accurate estimation algorithm, with a prediction accuracy of 96.89%. The random forest algorithm exhibited the highest accuracy in this study.

The third question aimed to predict student failure in the initial period by calculating the prediction coefficients of the model using the Random Forest model. The coefficients and a constant value of 0.076 were collected for the choices made by the students, and a score above 0.50 was considered successful. Based on this, a simple program was developed in Excel. The student’s total score is the sum of the fixed values across the responses to the variables in the program, and it predicts whether the student will be successful or unsuccessful. Furthermore, because it determines under what conditions the student can be successful, the deficiencies of a new student entering the system are eliminated in this process, and the system is used as an early warning system. It is assumed that the coefficient predictions can provide a system with additional information.

On the other hand, certain coefficients, such as the negative weight associated with PhD-level parental education, appear counterintuitive when compared to the prior literature. This anomaly can be explained by multiple factors. First, the sample size for these subgroups was relatively small, possibly leading to unstable predictions. Second, contextual dynamics, such as the pressure imposed by highly educated parents or their limited availability due to professional obligations, could inadvertently hinder student outcomes. We advise interpreting these specific coefficients with caution, as they may not reflect universal causal relationships, but rather localized or sample-specific effects.

The study was conducted using data collected from 613 students in the central district of Antalya. More generalizable results in terms of the assessment of students’ achievement levels can be obtained if a much larger number of study groups are selected for the research. It is not known whether the answers to the questions were given openly, as the data were collected online during the pandemic. The extent to which distance learning affects student success is unknown. Therefore, comprehensive analyses can be conducted in these two situations. The most accurate classification model can be clarified by working with other machine learning algorithms to evaluate student performance. The dataset is divided into three parts. By comparing the accuracy levels of the training, test, and validation sets, the results could be compared without cross-validation. The significance of these factors can be verified by collecting the data again and increasing the factors that influence student success. Students’ past performance can be assessed together with the results of this study by performing a logistic regression analysis of numerical values using the marks obtained in the oral and written examinations. Thus, the assessment of students’ future achievement status can be observed in two ways. When developing software for the study, students can fill in this form online and obtain information about their success. In the case of failure, the reasons for failure can be analyzed as an early warning system. In this way, a recipe can be created to help students achieve success. Therefore, this method may be more effective in this area.

It is believed that the model proposed in this study will make a significant contribution to teachers in the formative assessment process, and will have an impact on increasing student success in academic performance with the actions to be taken. It is also expected to provide an effective proposed solution in terms of cost and resources for educational policymakers through early identification and intervention of academic failure. It is expected that this developed model will help teachers meet students’ needs more quickly and ensure equal opportunities in the classroom. It is possible to intervene with a student who is academically unsuccessful during the process. Steps can be taken to ensure that students are academically successful. From a decision-maker’s perspective, integrating the system into educational processes will ensure a more efficient use of educational resources, and the failure rate per student will be significantly reduced owing to timely interventions. As the system is strengthened by data analysis and teacher feedback, it enables the modernization of education policy and the establishment of more effective decision-making mechanisms.

### Study limitations

This study has some limitations. First, the study was limited to data from students attending qualifying schools and schools closest to their homes in the central districts of a single province. The data collection process can be expanded by including data from all the provinces. In addition, student responses to the questionnaire developed in this study were limited by their ability to use digital resources.

This study only collected data from 1st grade high school and 8th grade middle school students; in this context, there are opportunities to work at different grade levels. Finally, the data collected were self-reported, which is a limitation due to the nature of the study. Although this study focused on base-level algorithms for transparency and interpretability, future research may benefit from experimenting with meta-ensemble strategies, such as voting or stacking, which have the potential to integrate the strengths of multiple models and enhance predictive performance in educational early warning systems.

This study relied on self-reported data, which may have been influenced by social desirability bias or recall inaccuracies. In addition, the sample was drawn entirely from the urban districts of a single province in Turkey, which potentially limits the generalizability of the findings. Future research should aim to replicate the model using a more diverse, nationally representative sample and explore the inclusion of behavioral or administrative data sources to reduce response bias.

A key limitation of this study lies in its external validation process, which was conducted on a relatively small sample of only 30 students. Although this provided a preliminary indication of the model’s generalizability, the restricted sample size substantially limits the statistical power and the reliability of the validation outcomes. To strengthen the robustness and applicability of future research, we strongly recommend the adoption of more rigorous validation strategies—such as k-fold cross-validation, stratified sampling, or the use of independent datasets drawn from diverse regions and educational contexts.These approaches would better support generalizable conclusions and provide stronger evidence for the broader applicability of early warning systems in diverse educational settings.

## Data Availability

The datasets generated and analyzed during the current study are available from the corresponding author upon reasonable request.
